# Hypoxia-Activated Alkylating Agents in BRCA1-Mutant Ovarian Serous Carcinoma

**DOI:** 10.7759/cureus.1517

**Published:** 2017-07-26

**Authors:** Michael Conroy, Mitesh J Borad, Alan H Bryce

**Affiliations:** 1 Cancer Center, Mayo Clinic, Scottsdale, AZ; 2 Haematology Oncology, Mayo Clinic, Scottsdale, AZ; 3 Hematology Oncology, Mayo Clinic, Scottsdale, AZ

**Keywords:** brca1 mutation, ovarian cancer, evofosfamide, th-302, alkylating agent, investigational new drug

## Abstract

Breast cancer 1 antigen (BRCA 1) and breast cancer 2 antigen (BRCA2) genes play a significant role in deoxyribonucleic acid (DNA) repair by means of interstrand crosslink repair, and deleterious germline mutations of these are responsible for most hereditary breast and ovarian cancers. Therapeutic strategies which specifically target interstrand crosslink repair can therefore be helpful in patients with harmful mutations. We describe two patients with advanced ovarian cancer and deleterious BRCA1 mutations who were treated with TH-302, a hypoxia-activated alkylating agent.

## Introduction

Breast cancer 1 antigen (BRCA 1) and breast cancer 2 antigen (BRCA2) are deoxyribonucleic acid (DNA) repair genes responsible for repairing interstrand crosslinks and double strand DNA breaks. They are best known for germline mutations which play a major role in hereditary breast and ovarian cancers (HBOC), and account for approximately 13% of ovarian cancers. Inheriting a single mutant BRCA2 allele confers a lifetime risk of breast cancer (45%; 31%-56%) and ovarian cancer (11%; 2.4%-19%) [1]. For these reasons, BRCA mutant breast and ovarian cancers have garnered considerable attention among investigators looking to develop targeted therapies. In addition, a significant proportion of sporadic cancers demonstrate acquired defects through this pathway and may benefit from similar treatment approaches.

The most popular therapeutic strategies that exploit DNA repair defects so far focus on the use of poly(ADP)ribose polymerase (PARP) inhibitors, which interfere with base excision repair and lead to the accumulation of DNA double-strand breaks [2]. However, more traditional chemotherapeutic agents can also exploit this pathway, including DNA alkylating agents. Alkylating agents aim to induce apoptosis by creating double strand DNA breaks that cannot be repaired by repair-deficient cell populations.

Herein we describe two patients with germline BRCA mutations who were treated with evofosfamide (TH-302), a hypoxia-activated alkylating agent, with a view to engage in tumor-selective targeting of the Fanconi anaemia (FA)/BRCA pathway. They were treated under separate, Food and Drug Administration (FDA)-approved, single patient investigational new drug (IND) protocols. The treatment was approved by the Mayo Clinic Institutional Review Board (IRB) and the FDA. Prior to treatment, an application for treatment was submitted to the US FDA Center for Drug Evaluation and Research. Each case was submitted separately, and after FDA approval, treatment was initiated using the previously established single agent dose for evofosfamide of 575 mg/m² given intravenously on a weekly schedule [3].

Evofosfamide is a hypoxia-activated prodrug consisting of 2-nitroimidazole linked to brominated isophosphoramide (Br-IPM), and is activated by cellular reductases in a hypoxic environment. Br-IPM forms DNA crosslinks that interfere with DNA replication and transcription. For cells to survive, these crosslinks must be excised and repaired [3]. 

The rationale for its use is the presence of a hypoxic microenvironment in tumors, which would theoretically lead to a targeted antineoplastic approach with less systemic toxicity than traditional cytotoxic agents. Once activated, the agent can also diffuse into surrounding oxygenated tumor cells and may be effective there via a ‘bystander effect’. Its efficacy so far has been investigated in soft-tissue sarcoma and metastatic pancreatic cancer, but the drug was not found to significantly improve survival in either setting [4-5].

## Case presentation

Patient 1

Patient 1’s family history was notable for her mother and grandmother dying from breast cancer in their 40s, and an additional maternal first cousin with breast cancer in her 50s. She also has a first cousin diagnosed with ovarian cancer at 50, and her father and his brother both suffered from prostate cancer. The patient’s niece was known to carry a deleterious BRCA mutation. Also to be noted, the patient herself had undergone a prophylactic bilateral mastectomy at the age of 39 years due to her family history of breast cancer.

Patient 1 presented at the age of 56 years with a three-month history of bloating and abdominal pain. She was taken to the operating room for presumed appendicitis, but she was instead found to have an adnexal mass with miliary disease. Her biopsy was positive for ovarian serous adenocarcinoma at multiple abdominal sites, stage III at time of diagnosis. She thereafter underwent an extensive resection, including modified radical hysterectomy, bilateral salpingo-oophorectomy, omentectomy, lymphadenectomy and cytoreduction with intraperitoneal catheter. Cancer Antigen-125 (CA-125) was 500 U/ml (normal 0 - 34.9) pre-operatively, and 205 U/ml post-operatively. Germline BRCA testing demonstrated a deleterious mutation of BRCA1: there was an E908X nonsense mutation.

**Figure 1:**
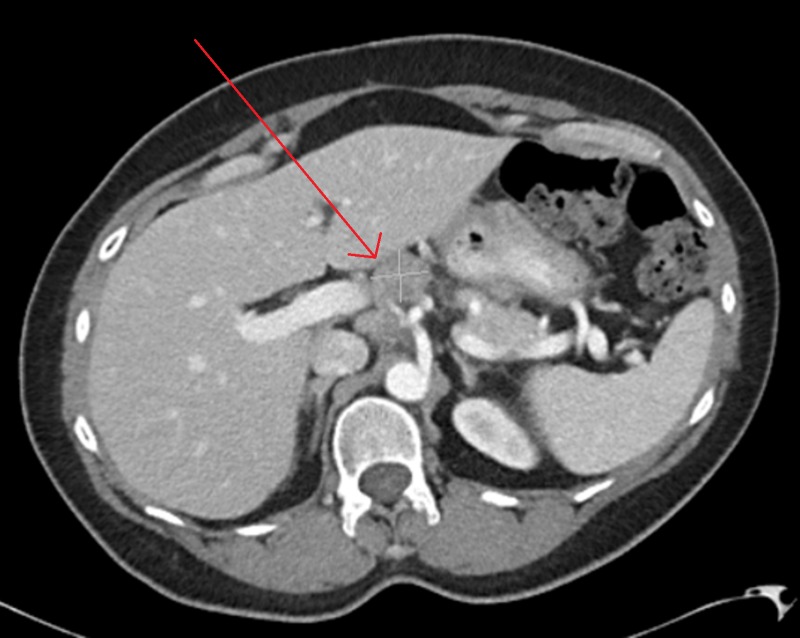
Abdominal CT of Patient 1 prior to starting evofosfamide, demonstrating a 2.5x2.8 cm target lesion, with CA-125 of 198 U/ml

**Figure 2:**
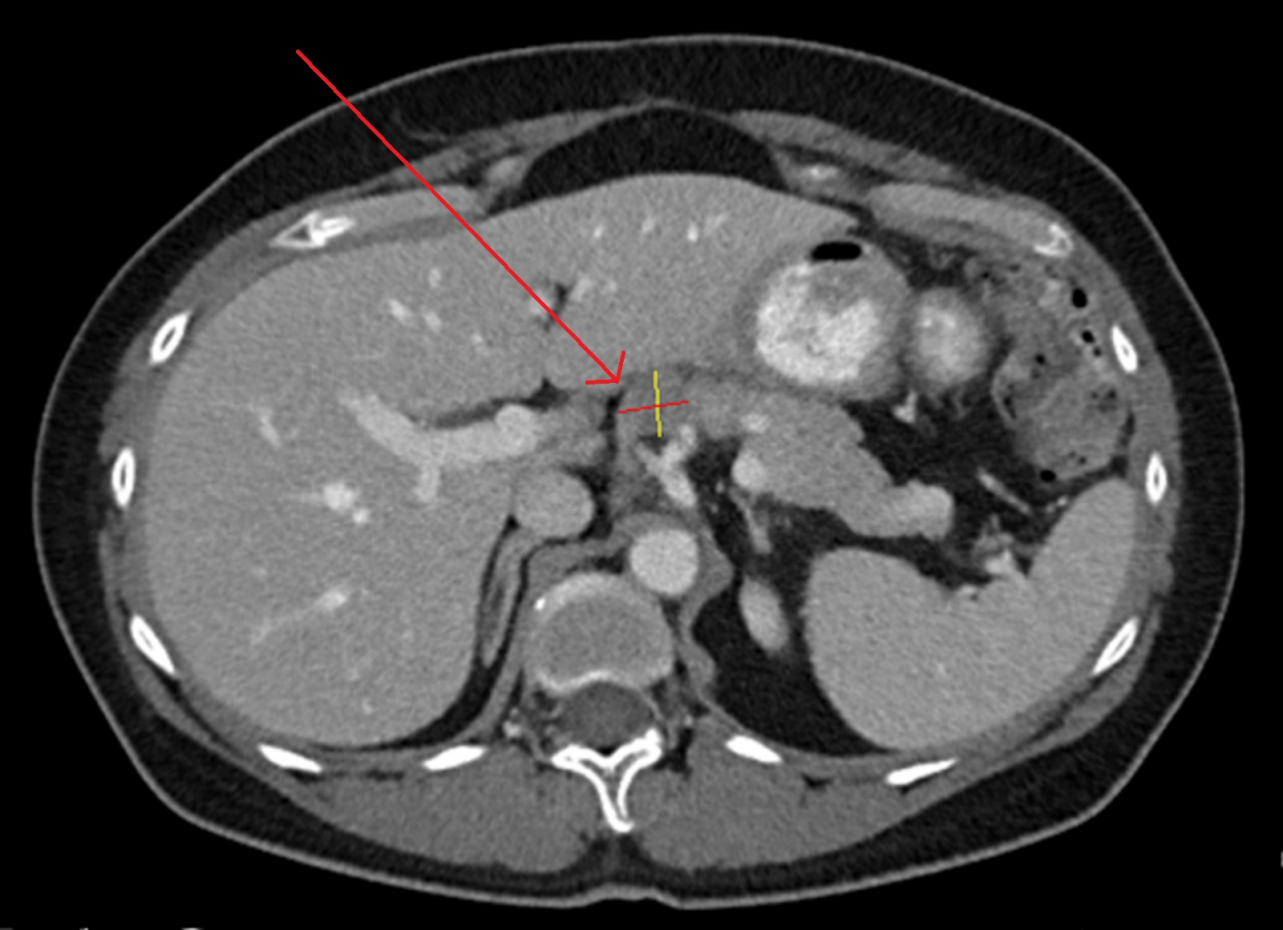
Abdominal CT of Patient 1 after three months of evofosfamide, showing a reduction in size of target lesion to 1.8x1.9 cm, with CA-125 62 U/ml

**Figure 3:**
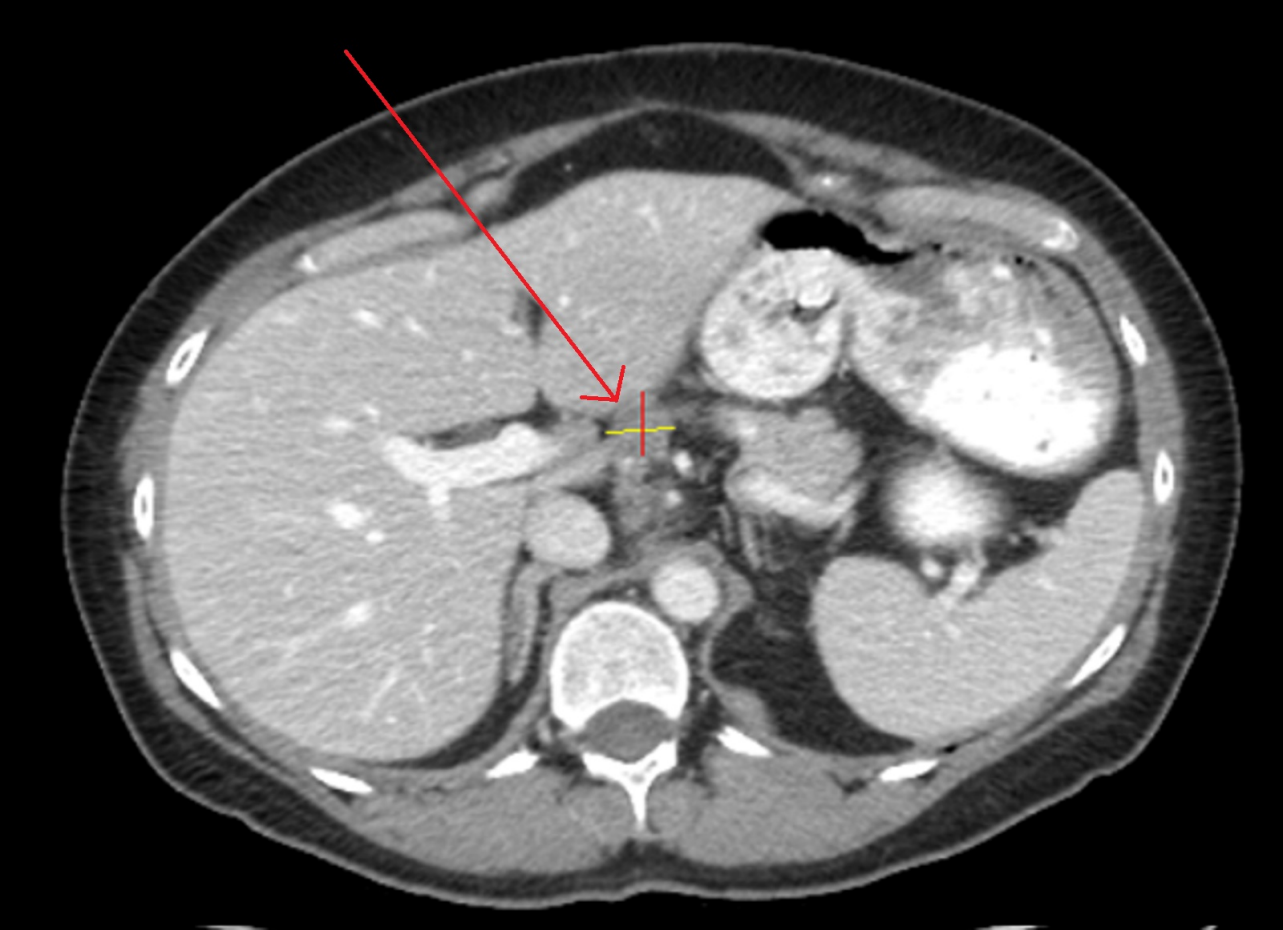
Abdominal CT of Patient 1 after completion of evofosfamide regimen, with stability in target lesion at 1.8 x 2.0 cm, with CA-125 107 U/ml

**Figure 4:**
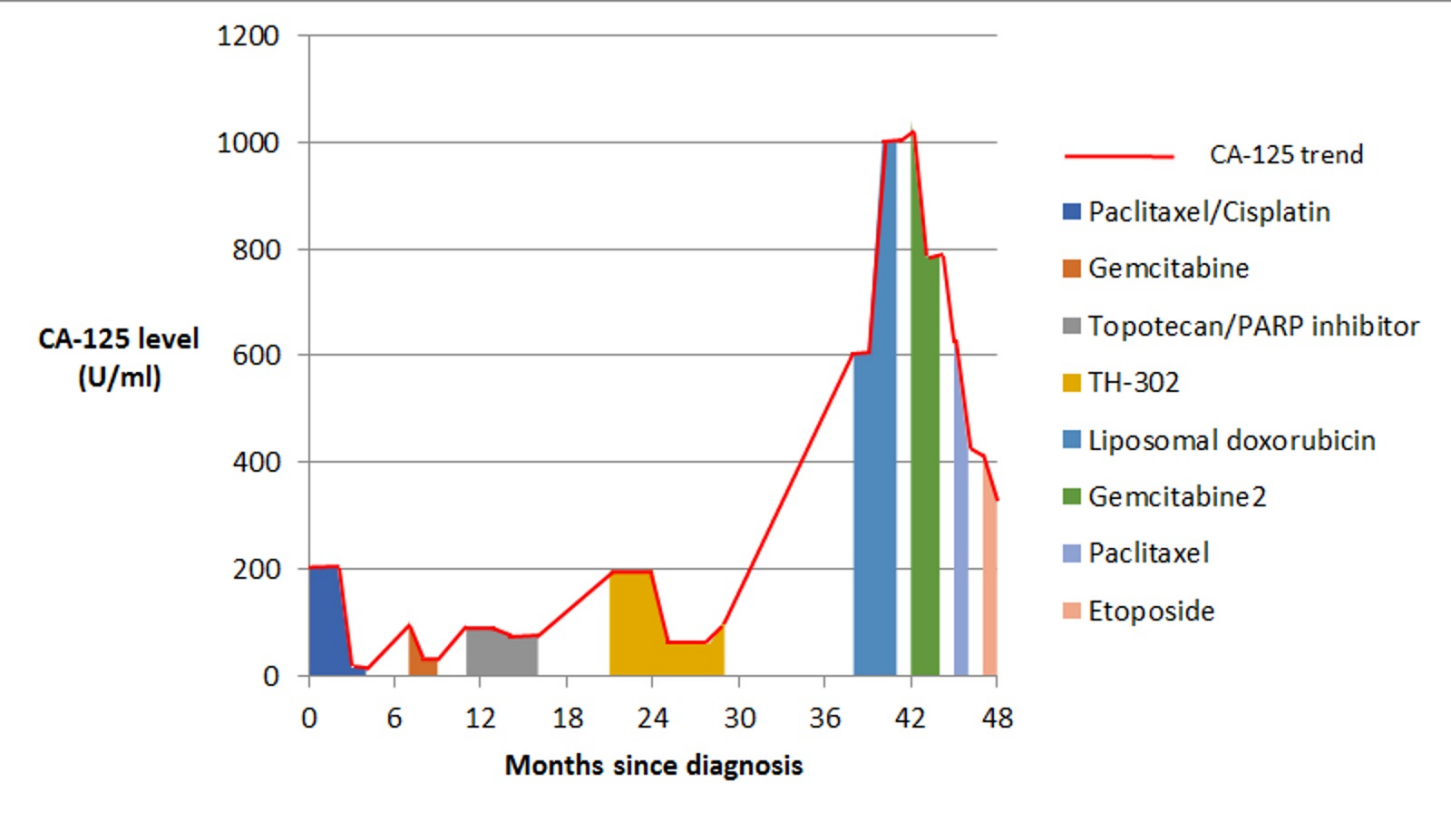
CA-125 trend in Patient 1 showing response to various treatment regimens

She was initially treated with six cycles of intraperitoneal (IP) and intravenous (IV) cisplatinum and paclitaxel (Table 1), after which there was no radiologic evidence of disease and cancer antigen 125 (CA-125) dropped to 16 U/ml. However, a second-look laparoscopy shortly thereafter identified persistent microscopic disease and CA-125 was noted to rise again shortly after. Therefore, she received four cycles of gemcitabine, which commenced three months after completing initial therapy. Gemcitabine was well-tolerated, with a drop in CA-125 from 104 U/ml to 16 U/ml. Abdominal recurrence was noted on imaging 11 months later, and she was therefore started on a clinical trial of topotecan and a PARP inhibitor, ABT 888, for seven cycles. CA-125 declined from 93 U/ml to 77 U/ml after two cycles, but it then increased to 198 U/ml by cycle 7. Computed tomography (CT) imaging after two cycles noted stable disease, but progression was noted in the abdominal lesions according to response evaluation criteria in solid tumors (RECIST) criteria after seven cycles (Figure 1-4).

**Table 1:** Patient 1 treatment regimens and response

Regimen	Treatment duration	Best radiographic response	Starting CA-125 (in U/ml)	Nadir CA-125 (in U/ml)	Duration of response (in months)	Grade 3 or 4 toxicities
Paclitaxel/cisplatin	6 cycles	Complete	205	16	(Residual disease identified on laparoscopy)	None
Gemcitabine	4 cycles	Complete	104	33	14	None
Topotecan/PARP inhibitor	7 cycles	Stable	93	77	6	None
TH-302	9 cycles	Stable	198	62	15	None
Liposomal doxorubicin	4 cycles	Progression	605	605	None	None
Gemcitabine	3 cycles	Progression	1040	786	None	None
Paclitaxel	2 cycles	Progression	636	423	None	None
Etoposide	4 cycles	progression	423	325	None	None

Patient 2

Patient 2 had a personal history of breast cancer at the age of 34, managed curatively with mastectomy. Her family history was significant for her sister dying of breast cancer at 34, her niece dying of breast cancer at 33, and an additional niece testing positive for BRCA1 mutation, who underwent risk-reduction surgery. Her mother, maternal aunt, and maternal grandmother were diagnosed with breast cancer at 46, 59, and 67 respectively. Her mother had bilateral breast cancer.

Patient 2 presented at 60 years of age with abdominal pain. A pelvic ultrasound identified an ovarian mass consistent with malignancy, and her CA-125 was 1,615 U/ml. Pathology confirmed high-grade serous ovarian carcinoma and the patient underwent a modified radical hysterectomy, bilateral salpingo-oophorectomy, omentectomy, and rectosigmoid resection with subcentimeter residual disease. She subsequently underwent BRCA1 sequencing that identified a deleterious mutation of the BRCA1 gene: the C1787S mutation, which consists of two adjacent missense mutations, C1787S and G1788D.

**Figure 5:**
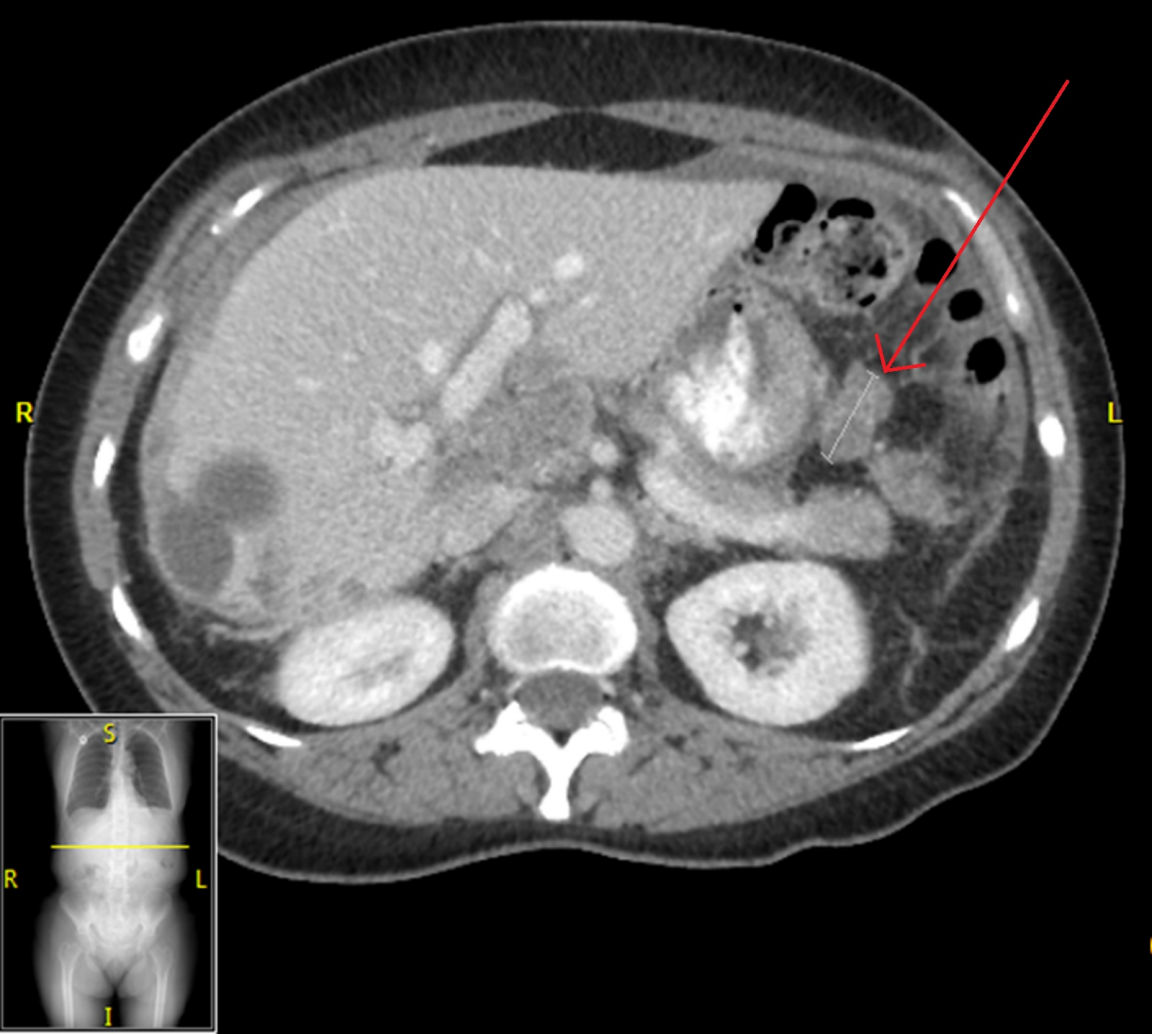
Abdominal CT of Patient 2 prior to starting evofosfamide, demonstrating target lesion of 2.7 cm in maximal dimension; CA-125 10,000 U/ml.

**Figure 6:**
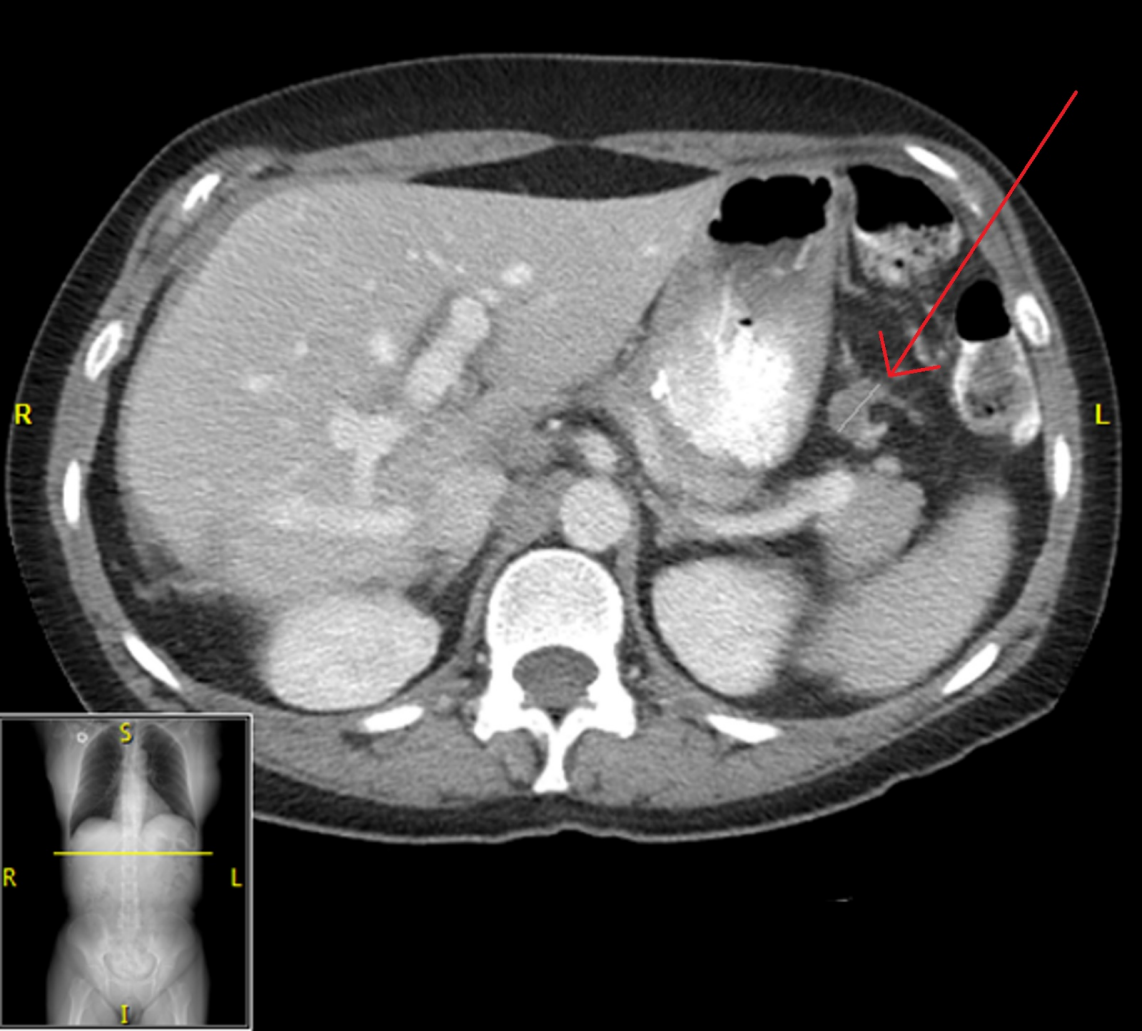
Abdominal CT of Patient 2 prior to cycle three of evofosfamide, showing a disappearance of peritoneal implants along with a reduction in size of target lesion to 1.8 cm in maximal dimension; CA-125 1602 U/ml

**Figure 7:**
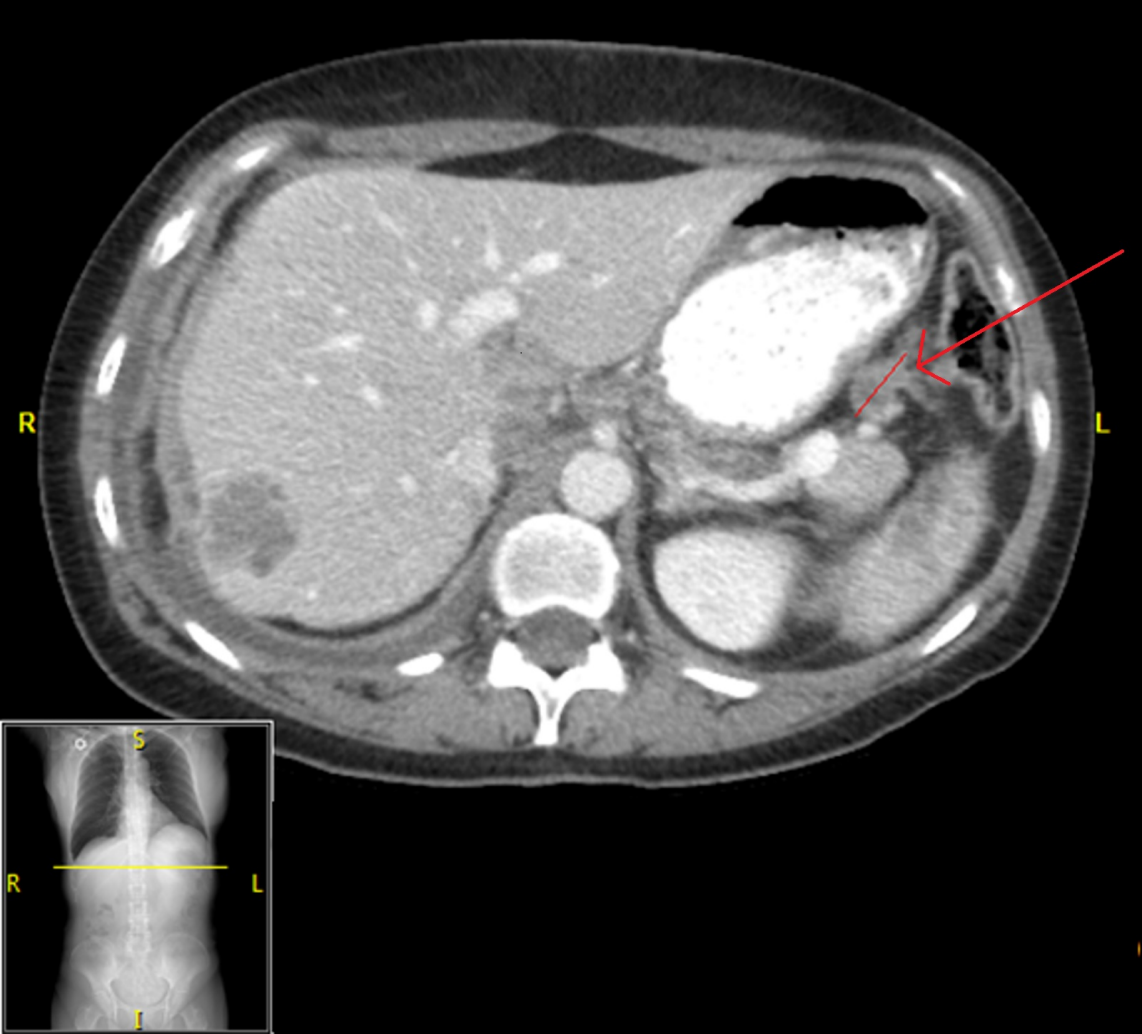
Abdominal CT of Patient 2 after cycle six of evofosfamide, showing an increase in size of target lesion to 2.3 cm in maximal dimension, with CA-125 6185 U/ml

**Figure 8:**
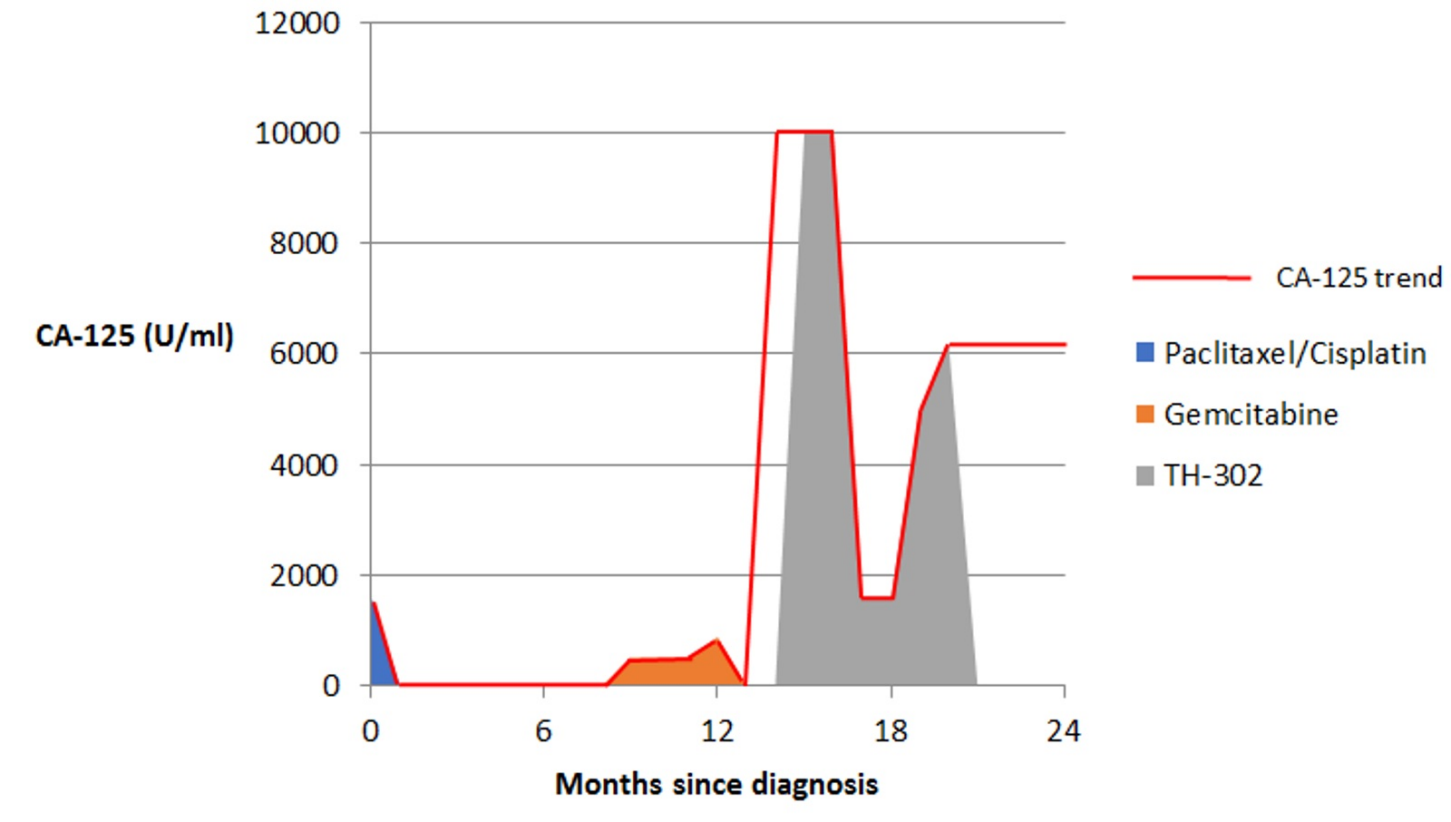
CA-125 trend in Patient 2 showing response to various treatment regimens

Her initial medical treatment combined intraperitoneal and intravenous chemotherapy with carboplatin and paclitaxel (Table 2) with a CA-125 nadir of 12 U/ml. Her CA-125 then increased to 512 U/ml six months later. She then received gemcitabine with no apparent response. Repeat staging after four cycles noted metastasis to liver and spleen and CA-125 rose to 883U/ml (Figure 5-8).

 

**Table 2:** Patient 2 treatment regimens and response

Regimen	Treatment duration	Best radiographic response	Starting CA-125 (in U/ml)	Nadir CA-125 (in U/ml)	Duration of response (in months)	Grade 3 or 4 toxicities
Paclitaxel / Carboplatin IV and IP	4 cycles	NA	1,615	12	3	Grade 3 nausea
Gemcitabine	4 cycles	Progression	512	512	None	None
TH-302	6 cycles	Partial response	10,000	1602	3	Thrombocytopenia, neutropenia

## Discussion

We describe two patients presenting with advanced-stage serous carcinoma of the ovary who, having failed conventional therapies, were enrolled in a single patient IND trial for the hypoxia activated pro-drug (HAP), evofosfamide. Both of the patients had deleterious BRCA1 mutations and developed a biochemical and radiographic response while on this regimen.

Hereditary breast and ovarian cancer (HBOC) syndrome is an autosomal dominant disorder that predisposes affected individuals to several early-onset tumors including breast, ovarian, prostate, and pancreatic cancer and melanoma. Most cases of HBOC are due to highly penetrant germline mutations in BRCA 1 and 2. These genes are responsible for a conserved DNA damage repair pathway, which repairs interstrand crosslinks and double strand DNA breaks through homologous recombination.

BRCA2 is also known as Fanconi Anaemia Group D1 (FANCD1) and is a member of the Fanconi anemia (FA) family of genes. The FA family is composed of 15 complementation groups that are mostly autosomal recessive in inheritance. The presence of one defective gene is considered a deleterious BRCA2 mutation, with a risk of HBOC. When both alleles of any component of the pathway are defective, patients develop the Fanconi phenotype. This phenotype includes a diverse range of physical abnormalities, developmental disabilities, and a high risk of malignancy. FANC and BRCA genes operate in concert to repair interstrand crosslinks and double-strand DNA breaks in the FA/BRCA pathway, and both FANC and BRCA proteins are required for high-fidelity repair. 

The FA/BRCA pathway has been approached as a therapeutic target from two perspectives: the use of PARP inhibitors and the use of agents targeting DNA crosslinking.

PARP is involved in base excision repair of DNA. Inhibition of PARP leads to the accumulation of DNA single-strand breaks, which can lead to DNA double-strand breaks at replication forks. In normal cells, these defects are repaired by means of error-free homologous recombination; however, in cells with deleterious BRCA1/2 mutations, there is deficient homologous recombination repair, and DNA double-strand breaks tend to accumulate. This pathway has been investigated for the treatment of triple negative breast cancer, prostate cancer, and ovarian cancer. Two PARP inhibitors currently have FDA approval: Olaparib has been approved for germline BRCA mutated (gBRCAm) advanced ovarian cancer that has received three or more prior lines of chemotherapy. An additional agent, niraparib, is approved for the maintenance treatment of adult patients with recurrent epithelial ovarian, fallopian tube, or primary peritoneal cancer who have shown complete or partial response to platinum-based chemotherapy. Unfortunately, neither was FDA-approved in the periods when Patient 1 and Patient 2 were ill.

More broadly speaking, PARP inhibitors have been developed to treat breast and ovarian cancer since experts believe that germline BRCA mutation should make a cancer more susceptible to DNA damaging agents through synthetic lethality. Synthetic lethality is an instance of potent and lethal synergy between two otherwise nonlethal events. It is now recognized that synthetic lethality in this setting should extend beyond BRCA1 and BRCA2 as targets, and beyond PARP inhibitors as agents. The two cases described here build on this concept and provide a theoretical framework to explore evofosfamide as a targeted therapy for patients with DNA repair defects.

The rationale for using DNA crosslinking agents is that there is a hypersensitivity to these agents in cells with defective interstrand crosslink repair.  It has long been noted that cells with FA deficiency are particularly sensitive to DNA crosslinking agents. Thus, the mainstay of diagnosis of FA has been measuring chromosome breakage in unbanded metaphase cells after treatment with diepoxybutane (DEB).

Similarly, BRCA1 mutant cells have been shown to have increased susceptibility to crosslinking agents, with response depending on the degree of BRCA expression. This was first shown in a laboratory setting by Tassone, et al. in 2003 [6]. Superior response to platinums in BRCA-mutant ovarian and breast cancer was shown as early as 2002 [7]. Conversely, it has been shown that upregulating BRCA1 leads to repair-mediated resistance to platinums [8]. Clinically, there is evidence of improved survival for ovarian cancer patients with a BRCA1 or BRCA2 mutation that may reflect this sensitivity to platinums; although breast cancer patients with a BRCA1 mutation had worse survival than non-carriers [9].

A significant body of work has also focused on sporadic breast and ovarian cancers with somatic BRCA1 dysfunction. Hypermethylation of the BRCA1 promoter is observed in up to 15% of sporadic ovarian cancers. Downregulation is typically attributed either to methylation of the BRCA1 promoter or up-regulation of microribonucleic acids (microRNAs) that target the BRCA1 transcript. Trials have mostly reported superior response to chemotherapy and improved survival among those with low BRCA1 expression [10].

With regard to our patients, both were managed on a regimen of evofosfamide based on the approach used in Phase 3 trials for sarcoma and pancreatic cancer. However, their dosing was higher than used in these trials (575 mg/m² vs 300 mg/m² and 340 mg/m², respectively). In addition, evofosfamide was used in combination with doxorubicin or gemcitabine in those trials, whereas it was used as a single agent in our patients.

Our two patients suffered from Stage IIIA epithelial ovarian carcinoma, which has a five-year life expectancy of 46%. Patient 1 and Patient 2 survived 59 months and 24 months from time of diagnosis, respectively. Both responded well to treatment with evofosfamide, showing stability and partial response by RECIST criteria, and significant biochemical responses. 

## Conclusions

The significance of BRCA1 and BRCA2 expression in malignancy is better recognized due to a better understanding of their role in sporadic cancers as well as in heritable disease. Targeting this pathway has had varying success in improving the quality of life and life expectancy in cancer patients. Further trials are needed that explore targeted therapy in patients with BRCA mutations.

In particular, impaired interstrand crosslink repair in BRCA1 mutants represents an interesting clinical target through the use of DNA crosslinking agents. We have described two patients with this mutation, suffering advanced ovarian cancer, and who showed a partial response or stable disease to a tumor-selective DNA-alkylating prodrug. 

Whether this response is primarily related to the presence of DNA repair defects is unknown. It may be that evofosfamide is generally active in ovarian cancer regardless of BRCA  mutation status. Nevertheless, the cases presented here are promising and support the further investigation of the effect of evofosfamide in ovarian cancer. More broadly, we propose that a tumor-agnostic genomically-driven study of DNA repair deficient tumors treated with evofosfamide or other DNA damaging chemotherapeutics should be pursued.  
